# Supplementary material for: Key Invariants in the Evolution of Sociality Across Taxa
Source: Biology (Basel). Author manuscript; Available in PMC 2025 Sep 17. (PMC7618134; doi:10.3390/biology14091239)
Supplement: Supplementary file 1 [file EMS208684-supplement-Supplementary_Materials.pdf]

Appendix A. Literature-Search Criteria & Inclusion Thresholds

We conducted a systematic literature search to ensure transparency and reproducibility. The following electronic databases were searched: Google Scholar, Web of Science, Scopus, and PubMed. The search covered publications from 1871 to 2025. Keywords included combinations of terms such as "quorum sensing," "allelopathy," "chemical communication," "plant volatile organic compounds", "microbial competition", "plant competition", "insect communication", "bacteria communication", "plants VOCs", "plant signaling", "cooperation", "social behavior" "aneural organisms" "social behavior genetic" "plant

behavior" and others. Only peer-reviewed articles published in English were considered. Studies were included if they provided empirical evidence. Reviews, meta-analyses, and conceptual papers were also included when they offered substantial synthesis or theoretical frameworks. Studies lacking sufficient methodological detail or relevance were excluded.
